# In the name of perseverance

**DOI:** 10.7554/eLife.92403

**Published:** 2023-09-05

**Authors:** Tanya Espino-Sanchez

**Affiliations:** 1 University of Utah Salt Lake City United States

**Keywords:** sparks of change, equity, inclusion, diversity, first-generation, grief, motherhood, mental health

## Abstract

Between honoring her immigrant family and making her children proud, a first-generation PhD student fights for her place in academia.

When I think of hard work and dedication, I think about my father and how, as a twenty-year-old undocumented immigrant, he was building a successful company that would end up employing more than 30 people. I was four at the time, and my sister had just been born. My parents were high school sweethearts who had met shortly after arriving in the United States from Mexico. I remember hearing my dad passionately pitch business ideas on the phone despite his limited English, and the dark circles under his eyes as he worked late nights and early mornings. I saw him fail, being told no, and pick himself back up every time.

These memories often kept me company during long days in the lab at the start of my PhD. For the first time in my life, I was states away from my family and the community that had lifted me up. I felt like I had walked into an alternative reality, one that I had been utterly unprepared for. My cohort was the most diverse in the history of my program, yet many faculty members weren’t used to being around people from various backgrounds. I received many comments questioning my place in the program, my future in science given that no one in my family has a PhD, and my legal status in the country.

I felt I had to prove to everyone that I deserved to be there, but the hardest part was believing it myself. I was constantly expecting to receive an email telling me that my admission had been a mistake. I had never experienced impostor syndrome with such intensity before: as an undergraduate at California State University of San Marcos, I had found a vibrant community of first-generation students who shared my struggles and my passion for making a difference. We were Latinx, female, a product of immigration, and we were doing science. Being around them motivated me to successfully apply for an NIH-funded program for undergraduates interested in research and to start leading initiatives to improve diversity in science. I had never thought that other scientific communities might not be so welcoming.

My husband was the only one who could understand that I was torn between wanting to stop my PhD, and knowing that I couldn’t. As a child of immigrants, you often have the responsibility to honor the sacrifices your family made for you, all while dealing with many of the same challenges. After they had fought so hard for my future, I couldn’t imagine telling my parents that I was quitting because I couldn’t handle someone questioning me. It was my turn to show that I could fight for my dreams and persevere in this environment.

I started to work overtime to prove that I belonged, and as I made the lab my home, I began to neglect my own. Too shy to ask for time off, I’d only visit my family for a few days here and there, my parents only making things worse by asking why I couldn’t stay longer. After a while I started to avoid going home or even spending too much time on the phone with them. In hindsight, this only made my depression worse, and I especially missed my little brother and sister from my mom’s second marriage. But the truth is, a part of me also enjoyed giving my time to research.

I pushed forward, and after a year I passed the exams required to continue with my PhD. I thought my strategy had finally paid off and now that I could focus on research, I started to make friends at work. We shared experiences about imposter syndrome, and I realized that I was not alone in my doubts. This helped to make failed experiments and obstacles feel like a normal part of science.

With things looking up, my husband and I decided we finally wanted to start a family. We had a plan: my mother would come live with us with my little siblings, who were 12 and 5 at the time, and we would support them while she would help us take care of the baby. For the first three months after I gave birth, everything seemed on track. My siblings were enjoying their new home and I returned to work knowing that my daughter was in safe hands with my mom. Seeing her and my dad become grandparents was one of the most beautiful things I’d ever experienced. Life was finally beginning to be brighter, until my mom became severely sick. Two months later, she passed away, during the middle of the pandemic. She was 44 years old.

My entire world came crashing down. In the midst of my grief, I thought about how I had fought so hard so she could watch me walk in my graduation gown after defending my PhD. I thought about every time I had said no to a family gathering, every time she had called and I had told her I was too busy to visit. These long nights in the lab to do experiments that always seemed to fail had not been worth the time I had missed with my family.

The darkness I felt losing her is indescribable, yet I had little space to grieve. Instead, I had to scramble to find care for my five-month-old baby while also fighting to adopt my siblings. As the pit of my depression grew deeper, I pulled away from everyone and everything that held me, including the connections I had made in my program. How could I explain my situation to my colleagues? No one would have been able to understand. To me, it was just another confirmation that academia was not made for someone like me, a first-generation Latina mother. I began to think about quitting again, but ultimately, I realized that I needed to prove to myself that I could survive this. Above all, I wanted to teach my now three children the values I had received from my family. Perseverance was not just about me pushing forward despite feeling like I didn’t belong; it was about fighting to make a place for myself, and for those who will come after me.

In retrospect, I believe that my journey would have been a little less dark and lonely if a researcher with a similar background had the time to sit with me and guide me. I wish someone could have shown me that PhD students like myself could make it, and that we do make a difference. Many people work towards improving diversity, representation and inclusion in academia – and rightly so – but in the process we shouldn’t forget about the consequences of bringing minorities into a system that was not designed for them.

I found my strength in the ‘village’ who rallied around me, from my husband and my dad to my 22-year-old sister who moved in with us and rearranged her entire life so I could go to the lab during normal hours, my paternal grandmother who came from Mexico every other month to help with the children, and the friends who flocked around us. This is where I had something to show for, the people who believed in me when I didn’t. With their love and support, I slowly made it back.

It’s been three years since my mother died, and my husband and I couldn’t be prouder of our family. My sister has now built a very successful career for herself, and the children are all flourishing. I know now that perseverance is necessary not just in research, but in all aspects of life, and that we must strive to have balance. As a scientist, I don’t feel like my impostor syndrome has diminished, but I’ve learned to fake it better. As long as we are all working towards improving the system for future generations, I can ignore those who are questioning my place.

I fell in love with science almost by accident, after curiosity drove me to take a few classes in college. I became fascinated with the idea that I could be the first to learn something new and then teach it to the world. Until then, science had been like a foreign language to me, something I thought only smart kids who were born speaking it could do. When I hear my three-year-old say that she wants to be a scientist like her mom one day, I know that my story matters. I will be graduating in a couple of months, and I can’t wait to share this moment with my village. In moments like these, I wish I could call my mom to tell her about all the exciting things happening in our lives.

## Share your experiences

This article is a Sparks of Change column, where people around the world share moments that illustrate how research culture is or should be changing. Have an interesting story to tell? See what we’re looking for and the best ways to get in touch here.

